# Our City Mortality

**Published:** 1881-10

**Authors:** 


### Editorial.

OUR CITY MORTALITY.—From the statistical tables furnished by the Health Department of this city, it appears that the gross mortality for July was 1,786, and for August 1,730. These figures not only indicate that the mortality for these months is as usual double that of any other two months of the year, but that their mortality is decidedly above the ordinary ratio for the same months in previous years. The increased mortality is as usual mostly from bowel affections in young children. But aside from this there were, in July, deaths from typhoid fever twenty-three, scarlet fever nineteen, diphtheria twenty-two, cerebro-spinal fever twenty-six, cerebral meningitis fifty, and small-pox fifty; and in August, from typhoid fever ninety-eight, scarlet fever fourteen, diphtheria forty-nine, cerebro-spinal fever thirty, cerebral meningitis forty-five, and small-pox 116. These figures, especially so far as they relate to typhoid fever, cerebro-spinal fever, and small-pox are extraordinary. Is the fault in the peculiarities of the season or in the sanitary condition of the city, or in both combined?

